# The Influence of the Electrodeposition Parameters on the Properties of Mn-Co-Based Nanofilms as Anode Materials for Alkaline Electrolysers

**DOI:** 10.3390/ma13112662

**Published:** 2020-06-11

**Authors:** Karolina Cysewska, Maria Krystyna Rybarczyk, Grzegorz Cempura, Jakub Karczewski, Marcin Łapiński, Piotr Jasinski, Sebastian Molin

**Affiliations:** 1 Faculty of Electronics, Telecommunications and Informatics, Gdansk University of Technology, ul. Narutowicza 11/12, 80-233 Gdansk, Poland; piotr.jasinski@pg.edu.pl (P.J.); sebastian.molin@pg.edu.pl (S.M.); 2 Department of Process Engineering and Chemical Technology, Chemical Faculty, Gdansk University of Technology, ul. Narutowicza 11/12, 80–233 Gdansk, Poland; maria.rybarczyk@pg.edu.pl; 3 International Centre of Electron Microscopy for Materials Science, Faculty of Metals Engineering and Industrial Computer Science, AGH University of Science and Technology, ul. A. Mickiewicza 30, 30-059 Krakow, Poland; cempura@agh.edu.pl; 4 Faculty of Applied Physics and Mathematics, Gdansk University of Technology, ul. Narutowicza 11/12, 80–233 Gdansk, Poland; jakkarcz@pg.edu.pl (J.K.); marcin.lapinski@pg.edu.pl (M.Ł.)

**Keywords:** alkaline electrolyser, electrocatalyst, electrodeposition, energy material, nanofilm, nickel foam, oxygen evolution reaction

## Abstract

In this work, the influence of the synthesis conditions on the structure, morphology, and electrocatalytic performance for the oxygen evolution reaction (OER) of Mn-Co-based films is studied. For this purpose, Mn-Co nanofilm is electrochemically synthesised in a one-step process on nickel foam in the presence of metal nitrates without any additives. The possible mechanism of the synthesis is proposed. The morphology and structure of the catalysts are studied by various techniques including scanning electron microscopy, X-ray diffraction, X-ray photoelectron spectroscopy, and transmission electron microscopy. The analyses show that the as-deposited catalysts consist mainly of oxides/hydroxides and/or (oxy)hydroxides based on Mn^2+^, Co^2+^, and Co^3+^. The alkaline post-treatment of the film results in the formation of Mn-Co (oxy)hydroxides and crystalline Co(OH)_2_ with a β-phase hexagonal platelet-like shape structure, indicating a layered double hydroxide structure, desirable for the OER. Electrochemical studies show that the catalytic performance of Mn-Co was dependent on the concentration of Mn versus Co in the synthesis solution and on the deposition charge. The optimised Mn-Co/Ni foam is characterised by a specific surface area of 10.5 m^2^·g^−1^, a pore volume of 0.0042 cm^3^·g^−1^, and high electrochemical stability with an overpotential deviation around 330–340 mV at 10 mA·cm^−2^_geo_ for 70 h.

## 1. Introduction

The oxygen evolution reaction (OER) has become the main limitation to the efficiency of the water splitting process [1,2]. In order to make the reaction more robust, novel anode catalysts, which would lower the overpotential needed to drive the reaction, are required [3]. Recently, both RuO_2_ and IrO_2_ have become highly active, benchmark catalysts as anode materials for the OER with an overpotential typically close to 350 mV at 10 mA·cm^−2^ [4]. However, their limited sources, high cost, and inferior stability at higher anodic potentials do not allow for large-scale usage [5,6]. Therefore, recently, there has been a huge effort to fabricate nanostructured oxide/hydroxide electrocatalysts based on earth-abundant elements [7,8,9]. Because of their eco-friendly properties and low cost, they have become interesting materials for different energy applications including batteries [10], supercapacitors [11], fuel cells [12], and alkaline water electrolyzers [13,14].

The traditional method for the synthesis of oxide/hydroxide films is based on the solid-state approach, which includes grinding and firing a mixture of certain metal oxides, nitrates, or carbonates [15,16]. Other possible chemical methods are sol-gel [17,18], combustion [19,20], and hydro/solvothermal routes [21,22]. However, all of the above-mentioned synthesis methods typically lead to the formation of pure catalyst powder, which requires further processing to produce an ink containing other additives such as a binder and conductive carbon powder [15,23]. This, in turn, may result in very poor stability of the OER electrode [24,25]. One of the promising, alternative synthesis techniques is electrodeposition. It allows for the formation of the catalyst directly on the conductive substrate without any additives [26,27]. Moreover, the properties of the deposited films can be easily tailored by changing the synthesis conditions such as the electrolyte concentration, type of solvent, and/or type of electrodeposition (potentiostatic/galvanostatic) [28]. By growing the catalysts directly on the substrate, their adherence and integrity, based on chemical bonding, should be superior to those of ink-based catalysts.

In the literature, there are several attempts to electrodeposit oxides/hydroxides based on different transition metals on conductive substrates [26,29,30,31,32]. Vigil et al. [33] reported the electrodeposition process for manganese oxide (MnO_x_) together with the conducting polymer 3,4-ethylenedioxytiophene (PEDOT) on a glassy carbon electrode for energy storage and conversion devices. Other studies proposed the electrochemical deposition of Mn_1.5_Co_1.5_O_4_ on Ni foam [31], Mn oxide rods on a gold/silica substrate [32], and Mn/Co or Ni/Co hydroxides on stainless steel [13,34] for supercapacitors.

Different kinds of electrodeposited oxides/hydroxides have also been studied as possible anode materials for the OER in alkaline environments [24,35,36,37,38]. A. Ramirez [35] studied the OER activity of differently electrodeposited manganese oxides such as MnO_x_, Mn_2_O_3_, and Mn_3_O_4_ on F:SnO_2_/glass. F. Yan reported the electrocatalytic properties of MnO_2_ on carbon cloth with an overpotential of 424 mV at 10 mA·cm^−2^ [24]. A lower OER overpotential of the electrode compared to a bare substrate was obtained for cobalt oxide synthesised on platinum, nickel, and iron [36,39,40]. P. Liu et al. [41] fabricated electrochemically etched α-cobalt hydroxide for the OER, characterising by an overpotential of 320 mV at 10 mA·cm^−2^.

Recently, significant effort has been made to synthesise mixed oxides/hydroxides based on Co [38], Mn [34], Ni, and Fe [37] due to their high electrocatalytic activity coming from their abundant defects and fast redox reactions [31]. It was shown that the OER overpotential could be decreased by introducing cobalt into oxides in different valence states [42]. It is also interesting to incorporate manganese into mixed oxides. It is cheap and environmentally friendly, and provides the possibility of several redox reactions due to its multi-valance state [29]. F. Yan presented the electrocatalytic properties of MnCo-layered double hydroxide (LDH) on carbon cloth, which exhibited an overpotential of 258 mV at 10 mA·cm^−2^ [24]. F.J. Perez-Alonso et al. [37] reported Ni/Fe-based oxides with different compositions on Ni foam and stainless steel. Zn/Co-based spinels such as Zn_x_Co_3_-xO_4_ on gold [38] and ZnCo_2_O_4_ on platinum [43] have also been developed for the OER. Their overpotentials were determined to be 330 mV and 390 mV at 10 mA·cm^−2^, respectively. Bao et al. [44] introduced Mn-Co based LDH-graphene composite, which revealed an OER overpotential of 330 mV at 10 mA·cm^−2^ and showed superior bifunctional water splitting activity. In another work, (Co, Ni)Mn-LDH nanosheets were fabricated on multi-wall carbon nanotubes for efficient OER. The material exhibited an OER overpotential of 300 mV at 10 mA·cm^−2^ and revealed excellent long-term electrocatalytic stability [45]. The previous work of Lankauf et al. [46] presented Mn_x_Co_3-x_O_4_ deposited on nickel foam with an OER overpotential of 327 mV at 10 mA·cm^−2^ and only slight material degradation after exposure to an alkaline environment for 25 h. Recently, Mn/Co-based film materials have also become promising catalysts in sealed-oxygen batteries for triggering oxygen-related anionic redox activity [47].

It is well known that the electrocatalytic properties of the material strongly depend on the type of the substrate. Studies have shown that the most suitable active metal for an alkaline electrolyser is nickel [37,48]. It is relatively cheap and chemically stable. The porous nature of nickel provides a much higher electroactive surface area compared to planar electrodes [48]. This in turn supports the ion/electron conduction between the catalyst and electrolyte, which results in higher OER activity of the electrode. It should be noted that there are almost no reports on electrodeposited Mn-Co-based oxides/hydroxides on a nickel substrate for alkaline electrolysers. Additionally, most of the performed studies were focused on investigating the properties of the catalysts after thermal treatment (spinels). There is a lack of literature reports on the structural and OER activity of as-deposited different forms of Mn/Co oxides/hydroxides without any post-treatment.

In this work, electrodeposited Mn-Co-based nanofilms on Ni foam were studied as potential catalysts for the oxygen evolution reaction. The catalysts were synthesised in a one-step process in an aqueous solution of manganese and cobalt nitrates without any additives. The possible mechanism of such a synthesis was proposed. The films were studied with regard to their structural and electrocatalytic properties for the OER. Mainly, the influence of the concentration ratio of Mn/Co nitrates used in a synthesis solution and deposition charge were investigated.

## 2. Materials and Methods

### 2.1. Chemicals and Materials

The chemicals used in this work were manganese (II) nitrate tetrahydrate (Mn(NO_3_)_2_·4H_2_O) (99%, Panreac), cobalt (II) nitrate hexahydrate (Co(NO_3_)_2_·6H_2_O) (98%, Sigma Aldrich, Saint Louis, MO, USA), and potassium hydroxide (KOH) (Stanlab, Lublin, Poland). The substrate was a pure nickel foam (~96% porosity, 110 pores per inch) or nickel foil. The solutions were prepared with distilled water (Millipore Elix Essential 3, Millipore corporation, Billerica, MA, U.S.A, >12 MΩ cm).

Before each electrodeposition process, the substrate was ultrasonically cleaned in distilled water and acetone, respectively for 5 min each, and dried in the air.

### 2.2. Preparation of Electrocatalysts

Mn-Co-based films were electrochemically synthesised in a one-step process at −1.1 V vs. Ag/AgCl in an aqueous solution of differently concentrated Mn(NO_3_)_2_·4H_2_O and Co(NO_3_)_2_·6H_2_O (≈pH 3) with the deposition time limited by charges of 60, 120, and 200 mC at 25 °C. The concentration ratios of Mn(NO_3_)_2_·4H_2_O to Co(NO_3_)_2_·6H_2_O were 2 mM to 4 mM, 2 mM to 6 mM, 2 mM to 8 mM, 4 mM to 2 mM, and 0 mM to 4 mM.

The experiments were performed in a one-compartment water-jacketed, three-electrode cell controlled by a VersaSTAT 4 potentiostat. The working electrode was nickel foam or nickel foil (0.5 cm × 0.5 cm) with a working area of 0.25 cm^2^. An Ag/AgCl in 3M KCl was the reference electrode (Hydromet) and a coiled platinum wire was the counter electrode.

### 2.3. Electrochemical Measurements

The electrochemical measurements were conducted in the same setup as the electrodeposition process (see Section 2.2) in an aqueous solution of 1 M KOH with a measured pH ≈ 13.9 at 25 °C. The temperature was controlled by a JULABO F12 thermostat. Before each measurement, the electrolyte was continuously purged with Ar for 20 min. The linear scan voltammetry (LSV) data were recorded from 0.1 to 1 V vs. Ag/AgCl with a scan rate of 5 mV·s^−1^. The electrochemical impedance spectra (EIS) were acquired in the frequency range of 10 kHz–0.1 Hz at 0.7 V vs. Ag/AgCl with an amplitude of 10 mV. The EIS data were fitted with the Zview software. The double layer capacitance (C_dl_) was determined based on the cyclic voltammetry (CV) performed by sweeping the potential across the non-faradaic region from 0.15 V to 0.25 V vs. Ag/AgCl at different scan rates (10, 20, 40, 60, 80, 100 mV·s^−1^) in an aqueous solution of 1 M KOH (Figure S1a,c,e, Supplementary content). The CV allowed for the determination of C_dl_ based on the following equation C_dl_ = i_dl_·(2ʋ)^−1^ = (i_a_ − i_c_)·(2ʋ)^−1^, where i_dl_ is the double-layer current density; i_a_ and i_c_ are the anodic and cathodic current densities, respectively; and ʋ is the scan rate. Thus, plotting half of the double-layer current density as a function of the scan rates yielded straight lines with slopes equal to the double-layer capacitance (Figure S1b,d,f, Supplementary content). The electrochemical surface area (ECSA) of the samples was determined according to the equation ECSA = C_dl_·A·C_spec_^−1^, where A is the geometric surface area of the sample and C_spec_ is the specific capacitance of 0.040 mF·cm^−2^_geo_, which is a typical value reported for a metal electrode in an aqueous alkaline solution [49]. The stability test was performed at 10 mA·cm^−2^ for approximately 70 h. Before each electrochemical measurement, CV of the studied material was carried out in order to obtain an activated and stable system. For this purpose, the CV was carried out from 0.1 to 0.6 V vs. Ag/AgCl with a scan rate of 100 mV·s^−1^ for at least 20 cycles.

All of the potentials were calibrated to the reversible hydrogen electrode potentials (vs. RHE) according to the equation E_vs. RHE_ = E_vs.Ag/AgCl_ + E^0^_Ag/AgCl in 3MKCl_ + 0.059 pH. Unless otherwise stated, the values of all potentials were iR-corrected to remove the effect of the solution resistance according to the equation E_iR-corrected_ = E_applied_ − iR_un_, where i is the current and R_un_ is uncompensated ohmic electrolyte resistance in an Ar-saturated 1 M KOH solution. The overpotential (η) for the oxygen evolution reaction was calculated by the following equation: η = E (10 mA·cm^−2^) − 1.23 V (vs. RHE) [50].

### 2.4. Material Characterisation

The morphology and elemental analysis of the catalysts synthesised under different conditions were investigated using a FEI QUANTA FEG 250 scanning electron microscope (SEM, Thermofisher (FEI), Waltham, MA, USA) with energy dispersive X-ray (EDX) analysis and with a FEI Titan G2-300 transmission electron microscope (TEM, Thermofisher (FEI), Waltham, MA, USA). For the TEM, the Mn-Co film was scratched off the nickel substrate.

X-ray photoemission spectroscopy (XPS, Omicron NanoTechnology, Taunusstein, Germany) measurements were carried out with Omicron NanoTechnology ultra-high vacuum equipment. The hemispherical spectrophotometer was equipped with a 128-channel collector. The XPS measurements were performed at room temperature at a pressure below 1.1 × 10^−8^ mBar. The photoelectrons were excited by an Mg-Kα X-Ray source. The X-ray anode was operated at 15 keV and 300 W. The results were corrected using the C1s peak (285.0 eV). The XPS spectra were analysed with the Casa-XPS software using a Shirley background subtraction and Gaussian–Lorentzian (GL30) curve as a fitting algorithm. XPS spectra were fitted with residual standard deviations (STDs) lower than 0.8 and 1.1 for the Co2p and Mn2p lines, respectively. 

X-ray diffraction (XRD, Bruker, Billerica, MA, USA) measurements were conducted on a Bruker D2 Phaser 2nd generation diffractometer with CuKα radiation (λ = 1.5404 Å). Data were collected from 2Θ = 10° to 90° with a step size of 0.01° at room temperature. XRD measurement was performed for optimised Mn-Co on nickel foil deposited from the solution of Mn/Co 2 mM/8 mM for 200 mC (before and after alkaline treatment in 1 M KOH).

The specific surface area of the samples was also characterised by a Brunauer–Emmett–Teller (BET, Micromeritics Instruments Corporation, Norcross, GA, USA) Micromeritics Gemini V apparatus (model 2365). For each measurement, the sample weight was in the range of 0.01–0.02 g. The degassing temperature reached 120 °C. The contact angle of the electrodes was determined by an OCA15 goniometer. The liquid used was distilled water. The non-local density functional theory (DFT) model was applied in order to assess the pore characteristics. The pore volume was calculated using the normal liquid density of the adsorbate [51].

## 3. Results and Discussion

### 3.1. Electrochemical Formation and Morphology of Mn-Co-Based Oxides/Hydroxides on Ni Foam

**Figure 1:**
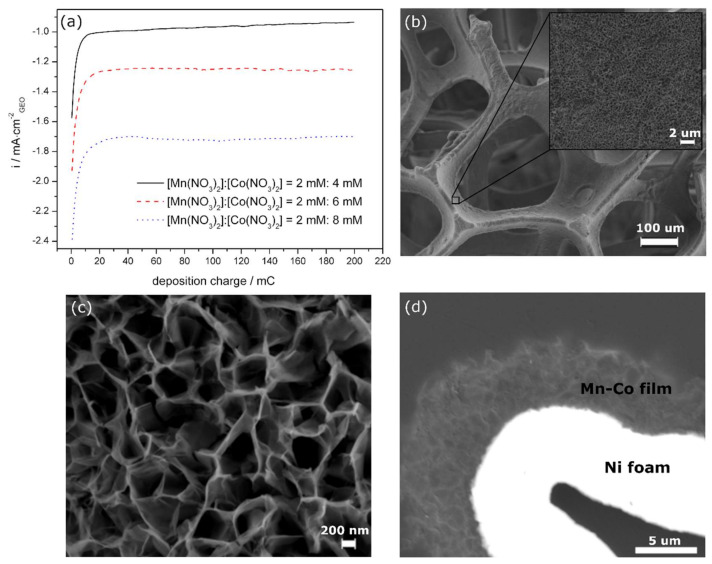
(**a**) Synthesis graphs recorded during the electrochemical synthesis of Mn/Co oxide/hydroxides at −1.1 V vs. Ag/AgCl in an aqueous solution of differently concentrated Mn(NO_3_)_2_·4H_2_O and Co(NO_3_)_2_·6H_2_O with the deposition time limited by a charge of 200 mC; (**b**–**d**) SEM images of Mn-Co film deposited on nickel foam in a solution of Mn/Co (2 mM/8 mM) for 200 mC with different magnifications (**b**,**c**—surfaces, **d**—polished cross-section).

All manganese–cobalt-based films (Mn-Co) were electrochemically synthesised in a one-step process in an aqueous solution of differently concentrated Mn(NO_3_)_2_·4H_2_O (Mn) and Co(NO_3_)_2_·6H_2_O (Co) without any additives. The synthesis graphs recorded during the electrodeposition are presented in Figure 1a. 

At the beginning of the deposition, the cathodic current density decreases very fast, indicating the growth of a new phase on the nickel substrate [13]. After a certain time, when a total charge of ~10 mC is reached for all of the samples, the current density becomes stable, due to the steady state formation of Mn-Co deposits. The synthesis trend is similar for all studied cases. A higher value of the cathodic current density is found for the higher concentrations of Co(NO_3_)_2_·6H_2_O in the synthesis solution. Moreover, the current density during the steady-state deposition increases linearly with a higher cobalt concentration in the solution. For comparison, Co and Mn-Co films were deposited on nickel foam in the presence of only 4 mM Co(NO_3_)_2_·6H_2_O (Mn/Co 0 mM/4 mM) or in the presence of a higher content of manganese compared to cobalt, i.e., 4 mM Mn(NO_3_)_2_·4H_2_O/2 mM Co(NO_3_)_2_·6H_2_O (Mn/Co 4 mM/2 mM). The synthesis curves recorded for such cases reveal that the deposition process in the presence of cobalt alone proceeds much more slowly (lower cathodic current density) compared to the synthesis in the presence of both Mn and Co (Figure S2, Supplementary content). The maximum cathodic current density for Mn/Co 0 mM/4 mM and Mn/Co 2 mM/4 mM was determined to be approximately −0.22 mA·cm^−2^_geo_ and −1 mA·cm^−2^_geo_, respectively. Moreover, the current density achieved during the deposition of Mn-Co in the presence of a higher concentration of manganese was determined to be −0.33 mA·cm^−2^_geo_, which is much lower compared to Mn-Co with a higher content of cobalt (Figure S2, Supplementary content). These all indicate that the nucleation rate is the fastest in the presence of both Mn(NO_3_)_2_·4H_2_O and Co(NO_3_)_2_·6H_2_O with a higher content of cobalt in the ratio.

The as-deposited Mn-Co films were characterised by a brownish/gold and green colour, indicating the presence of manganese and cobalt oxides/hydroxides, respectively. Here, it should also be noted that soaking the as-prepared samples in an aqueous solution of 1 M KOH resulted in a changing of the catalyst’s colour from brownish/gold-green to black, suggesting a change in the catalyst’s structure (Figure S3, Supplementary content). Because of that, it was important to also study the properties of the films after alkaline treatment in 1 M KOH, which will be related to the properties during the measurements. 

The morphology of the differently prepared samples was studied by scanning electron microscopy (SEM). Figure 1b–d present SEM images of Mn-Co films on nickel foam deposited in an aqueous solution of Mn/Co 2 mM/8 mM with the deposition time limited by a charge of 200 mC. The SEM analysis confirmed the successful deposition of the Mn-Co-based film on the porous nickel foam (Figure 1b,c). The structure of the as-deposited Mn-Co films was characterised by interconnected nanoflakes uniformly deposited on the nickel substrate, forming a porous interconnected 3D network. The thin nanoflake structure was also observed for a chemically synthesised Mn-Co catalyst [44]. Such a morphology might support the fast transport of hydroxide ions (OH-) due to the easily accessible open spaces [30]. This, in turn, should ensure high structural stability in the OER [24]. There was no significant change in the surface topography between the Mn/Co films synthesised in the aqueous solutions of Mn/Co 2 mM/4 mM, 2 mM/6 mM, and 2 mM/8 mM (Figure S4, Supplementary content). A similar nanosheet structure was obtained when the substrate was nickel foil (Figure S5a, Supplementary content).

The structure of the catalyst after synthesis in the presence of only 4 mM Co(NO_3_)_2_·6H_2_O revealed mainly a nanosheet-like structure, which in some places agglomerated (Figure S6a, Supplementary content). The film consisted only of the cobalt compounds, which was proven by the EDX elemental analysis (not shown here). A totally different morphology was observed for the Mn/Co film deposited in the presence of a higher concentration of Mn compared to that in cobalt Mn/Co 4 mM/2 mM (Figure S6b, Supplementary content). Here, the structure was characterised by randomly distributed and much smaller nanosheets with star-like structures with an atomic ratio of Mn to Co of 3.7:1. The results indicate that the nanosheet structure of the as-deposited Mn/Co film prepared in the solutions of Mn/Co 2 mM/4 mM, 2 mM/6 mM, and 2 mM/8 mM is mainly determined by the Co(NO_3_)_2_·6H_2_O.

The thickness of the deposited catalysts was determined based on the cross-sectional SEM images and varied from 2 to 7 μm for Mn-Co 2 mM/8 mM electrodeposited with a charge of 200 mC on nickel foam. Figure 1d presents a cross-sectional SEM image of the thickest part of the film obtained on nickel foam. For comparison, the same Mn-Co film was electrodeposited on nickel foil. The cross-sectional SEM image shows that the catalyst was homogenously deposited on the foil (Figure S5b, Supplementary content). The thickness of the film was determined to be approximately 2 μm. The irregular deposition of the catalyst on the nickel foam might be either due to its highly porous nature or the chosen deposition method. In the case of such a substrate, the efficiency of the electrodeposition might differ due to the difficulties of introducing the synthesis solution into the cavities of the foam, which is not the case when the planar electrode is used. The thickness of the Mn-Co film was virtually the same for the film synthesised in the presence of differently concentrated Mn/Co nitrates in the solution. 

The mass of the catalyst synthesised with the limited charge of 200 mC was determined to be around 50 μg for each studied case (based on a weight gain measurement on a microbalance). The theoretical mass was estimated based on Faraday’s law and was determined to be around 186 μg, assuming that the as-deposited film consists mainly of an LDH structure of CoOOH and MnOOH, with a total molar mass (M) of 179.87 g·mol^−1^, an electron loss (z) of 2, and a deposition charge of 200 mC. The differences in the experimental and theoretical mass are mainly due to the assumptions, which can greatly influence the theoretical calculations. It also indicates that the deposition process did not proceed with 100% efficiency.

### 3.2. XRD, XPS, and TEM Analysis of Mn-Co-Based Nanofilm

**Figure 2:**
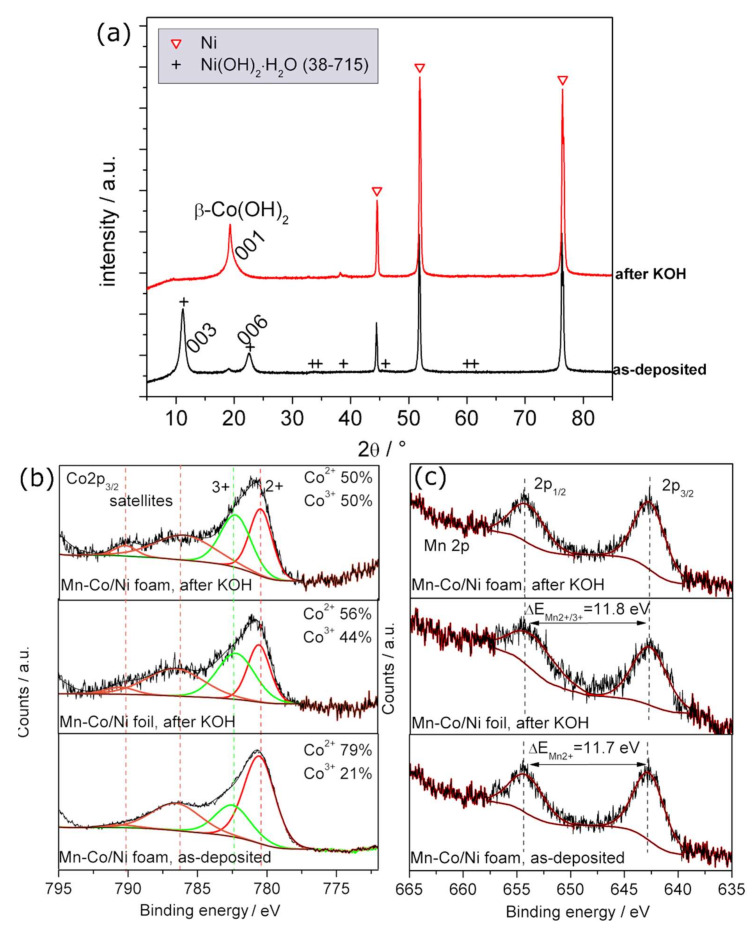
(**a**) XRD patterns of Mn-Co deposited on nickel before (red, triangle), and after (black, cross) alkaline treatment. The most intense diffraction peaks are assigned to Ni foil. XPS scans of (**b**) Co and (**c**) Mn of as-deposited and after-alkaline-treatment Mn-Co film. For XRD and XPS, the film was synthesised in a solution of Mn/Co 2 mM/8 mM for 200 mC on nickel foam/foil.

The structure of the as-deposited Mn-Co film (2 mM/8 mM) on nickel foil and after alkaline treatment in 1 M KOH was analysed by X-ray diffraction (XRD), and the results are presented in Figure 2a.

The peaks at 2θ of around 45°, 52°, and 76° associated with the presence of nickel are observed in each studied case, which is in agreement with the literature [52]. The rest of the peaks observed for the as-deposited Mn-Co film can be attributed to nickel hydroxide in accordance with the JCDS database (38–715). The presence of both planes (003) and (006) at 2θ of 10.8° and 22.5°, respectively, was related to the presence of α-phase cobalt hydroxide Co(OH)_2_ [34,53,54], which in the literature was assigned to the typical pattern of layered double hydroxides (LDH) [52,55]. Soaking the as-deposited Mn-Co film in 1 M KOH results in the formation of crystalline β-phase cobalt (II) hydroxide Co(OH)_2_, which can be clearly seen in the XRD spectra as a peak at around 19.3° [54]. Such a peak can also be observed for the as-deposited sample but with a much lower intensity. Comparing the results with the Pourbaix diagram, it can be noted that in a solution of alkaline pH, the formation of Co(OH)_2_ is expected.

The XPS analysis (Figure 2b,c) reveals the presence of the manganese and cobalt elements in the deposited film. In the Co 2p spectra (Figure 2b), two kinds of cobalt species are observed for each studied case, Co^2+^ and Co^3+^, at binding energies of 780.5 eV and 782.5 eV, respectively [52,56]. The Mn 2p spectra (Figure 2c) are split into Mn 2p_1/2_ and Mn 2p_3/2_, which are located at 653.5 eV and 641.8 eV, respectively, for the as-deposited film, indicating the presence mainly of Mn^2+^ [57,58]. After the alkaline post-treatment of the sample in KOH solution, the energy changes at around 11.8 eV, indicating the presence of both Mn^2+^ and possibly Mn^3+^ [57]. The percentage contents of certain elements in the as-deposited film and the film after alkaline treatment are presented in Table 1.

The analysis shows that the content of Co^2+^ to Co^3+^ changes in the Mn-Co film after alkaline treatment, indicating the oxidation of the Co^2+^ to Co^3+^. The post-treated Mn-Co/nickel reveals the possible presence of both Mn^2+^ and Mn^3+^, which indicates an oxidation of manganese due to the alkaline environment. It should be noted that in the case of manganese, the differences in binding energy related to certain valence states are very small, which cannot be interpreted with 100% accuracy based only on the XPS analysis. This is also the reason why in the literature, the same energy can be associated with different valence states of manganese in the film [52,59].

The content of the Co^2+^, Co^3+^, and Mn^2+/3+^ elements in Mn-Co film after alkaline treatment deposited either on nickel foam or nickel foil is virtually the same, indicating the good reproducibility of both the applied synthesis method and the post-treatment process in 1 M KOH.

**Figure 3:**
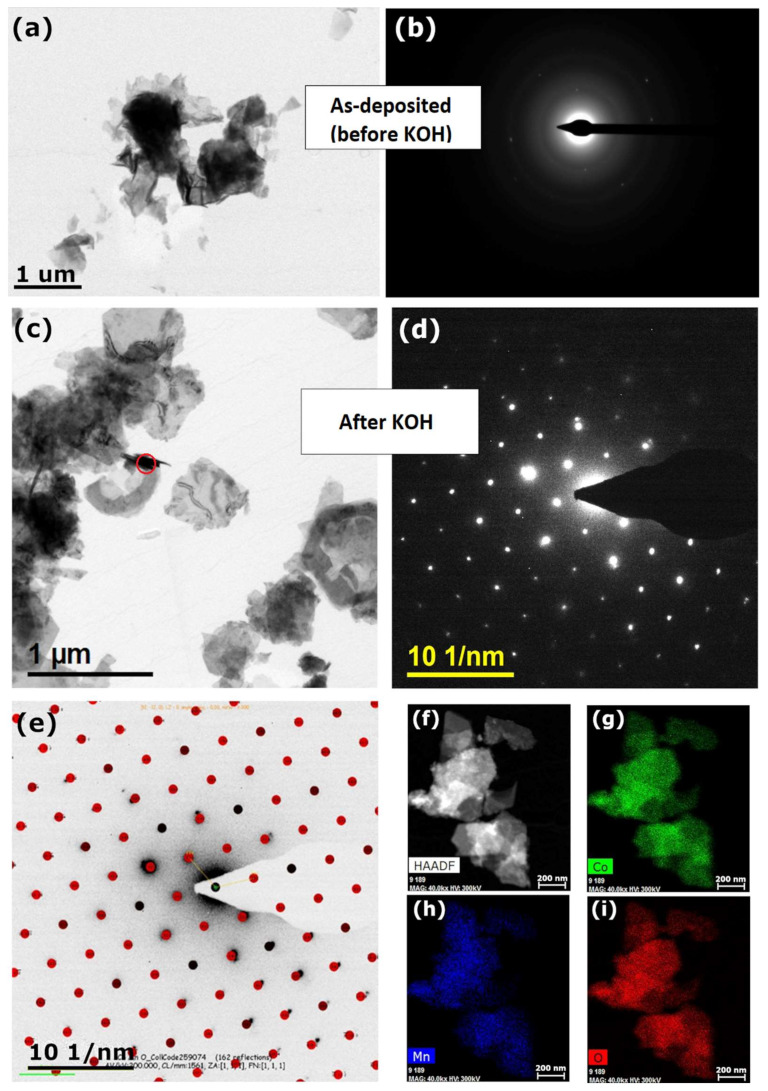
(**a**) Bright Field (BF) TEM image, and (**b**) corresponding experimental Selected Area Diffraction (SAED) pattern of Mn-Co film before alkaline treatment; (**c**) BF-TEM image, area of SAED pattern is marked with red circle, and (**d**) corresponding experimental SAED pattern from the area, marked on a BF-TEM image with a red circle, of Mn-Co film after alkaline treatment; (**e**) Experimental SAED pattern of area marked with a red circle, superimposed with simulated, theoretical diffractogram from cobalt manganese (IV/VI) oxide (0.25/1.75/4), (259073-ICSD) Zone Axis [111]; (**f**–**i**) TEM elemental mapping images of (**g**) cobalt, (**h**) manganese, and (**i**) oxygen of the Mn-Co film. Mn-Co film for TEM analysis was synthesised in an aqueous solution of Mn/Co 2 mM/8 mM (200 mC).

The morphology and microstructure of the Mn-Co film on nickel foam before (Figure 3a,b) and after (Figure 3c–e) alkaline treatment in 1 M KOH were also studied by transmission emission microscopy (TEM).

As can be seen from Figure 3a,b, the as-deposited film is characterised by the nanosheet, a mainly amorphous structure, which partially crystallised due to the formation of Co(OH)_2_ (Figure 2a). The crystallinity of the deposits increases upon alkaline treatment in KOH. The hexagonal platelet-like shape structure appeared after soaking the material in KOH solution (Figure 3c), which is a typical morphology for layered double-hydroxides [55]. The crystalline structure of the post-treated film is also proven by the Selected Area Diffraction (SAED) analysis (Figure 3d,e). The analysis of the experimental SAED pattern confirms the presence of crystalline particles of cobalt manganese (IV/VI) oxide (0.25/1.75/4). The results obtained by TEM are in agreement with the previous SEM, XPS, and XRD analyses (Figure 1 and Figure 2). The TEM elemental mapping images of the post-treated Mn-Co film confirms the successful synthesis of the Mn-Co-based film (Figure 3f–i). The analysis demonstrates that the film is composed of uniformly distributed cobalt (47.4 at.%), manganese (6.6 at.%), and oxygen (46 at.%). Thus, the atomic ratio of Mn to Co for the film synthesised in an aqueous solution of Mn/Co 2 mM/8 mM (200 mC) equals around 1:7.2. The atomic ratios of Mn to Co for 2 mM/4 mM and 2 mM/6 mM Mn/Co in the synthesis solution were determined to be 1:3.1 and 1:9.5, respectively. This indicates that the atomic ratio of Mn to Co in the film was not linearly related to the concentration of both the cobalt and manganese nitrates used in the synthesis solution.

**Figure 4:**
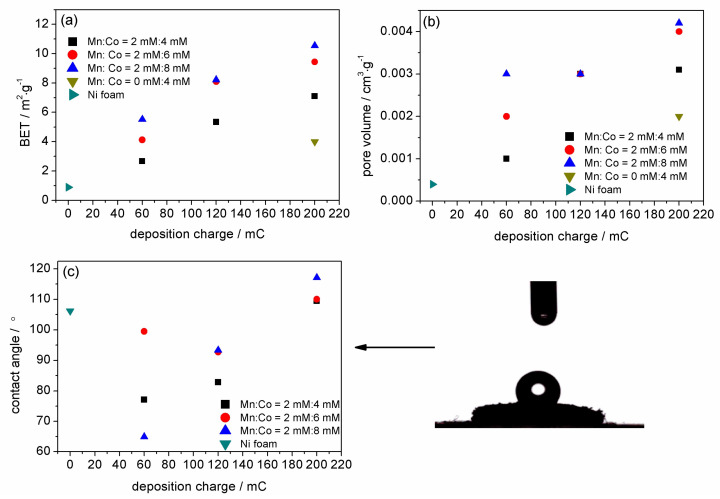
Evolution of (**a**) Brunauer–Emmett–Teller (BET) specific surface area, (**b**) pore volume, and (**c**) contact angle with an example of an optical microscopy image taken during the measurement of Mn/Co 2 mM/8 mM for bare nickel foam and nickel foam coated with Mn-Co film synthesised under different conditions as a function of the deposition charge.

The specific surface areas of the films were analysed based on the Brunauer–Emmett–Teller (BET) method. Figure 4 presents the evolution of the BET-specific surface area (a) and pore volume (b) determined for bare nickel foam and nickel foam coated with a Mn-Co film synthesised under different conditions as a function of the deposition charge (Qd). 

The graph clearly shows that the BET surface area increases gradually with both a higher content of cobalt with respect to manganese nitrates in the synthesis solution and with the deposition charge. Even though the higher concentration of cobalt versus manganese nitrates in the synthesis solution did not induce any significant changes in the film morphology in the SEM images (Figure S4, Supplementary content), it strongly influenced the level of the specific surface area. Probably, the higher content of cobalt induced the formation of additional nanosheets, forming a percolation network with an open porous structure, which resulted in the higher specific surface area [29]. A higher surface area was also observed in the case of the higher deposition charge. This can be explained by the fact that a higher deposition charge for the same geometric area of the electrode results in a thicker deposited film [60]. This in turn, led to a higher specific surface area, which could be seen for each studied case in Figure 4a.

The evolution of the BET surface area also shows that each of the Mn-Co films prepared in solutions of Mn/Co 2 mM/4 mM, 2 mM/6 mM, and 2 mM/8 mM reveal a higher specific surface area compared to bare nickel foam. The highest surface area of 10.5 m^2^·g^−1^ was obtained for the Mn-Co film prepared in the solution of Mn/Co 2 mM/8 mM for 200 mC, which is almost 12 times higher in comparison to that of bare nickel foam.

The specific surface area of the film on nickel foam synthesised only in the presence of 4 mM cobalt nitrate with 200 mC was determined to be 4 m^2^·g^−1^, which is 2.6 times lower than that of the film synthesised in the presence of both manganese and cobalt compounds. 

The evolution of the corresponding pore volume for bare nickel foam and nickel foam coated with a Mn-Co film synthesised under different conditions as a function of the deposition charge is presented in Figure 4b. The trend is very similar to the trends obtained from the BET surface area, i.e., the pore volume of the film on nickel foam increases with an increasing deposition charge and with a higher content of cobalt in the Mn/Co concentration ratio in the solution. A higher pore volume indicates a more porous catalyst. The Ni foam coated with the Mn-Co film reveals a higher pore volume compared to bare nickel. Mn-Co-based nickel foam synthesised in the presence of Mn/Co 2 mM/8 mM (200 mC) exhibits the highest value of pore volume of 0.0042 cm^3^·g^−1^, which is 10.5 times higher in comparison to that for bare nickel foam.

The wettability of the surface of the electrode is also an important parameter with respect to OER activity. A more hydrophilic surface supports the detachment of the gas bubbles that are produced during the water electrolysis process [6]. This, in turn, influences the electrocatalytical activity and stability of the catalyst in the OER process. Figure 4c presents the evolution of the contact angle for a Mn-Co film on nickel foam as a function of the deposition charge. Firstly, it can be observed that the deposition of the film for 60 mC for each studied case results in a decrease of the contact angle in comparison to that of bare nickel foam. This indicates that a thin layer of the catalytic material significantly increases the hydrophilicity of the electrode. The lowest value is obtained for the film synthesised in the solution of Mn/Co 2 mM/8 mM. A clear increase in the contact angle is observed for the higher deposition charge, which suggests that a thicker layer induces hydrophobicity of the surface. Moreover, it can be seen that a higher content of the cobalt in the Mn/Co ratio tends to be more hydrophobic with an increasing deposition charge. In addition to the changes in the film composition, the crystallographic phases present at the catalyst surface can also contribute to the evolution of the contact angle.

### 3.3. Probable Synthesis Mechanism for the Electrodeposition of Mn-Co Film on Nickel

The possible electrodeposition mechanism of the formation of the Mn-Co film on nickel in an aqueous solution of Mn(NO_3_)_2_·4H_2_O and Co(NO_3_)_2_·6H_2_O can be described as follows: After applying a potential of more than −1 V vs. Ag/AgCl at the nickel surface, both the reaction of the nitrate NO_3_^−^ ions (Equation (1)) coming from the Mn and Co compound, and the hydrogen evolution reaction (Equation (2)) can take place [29,34].

NO_3_^−^ + H_2_O + 2e^−^ → NO_2_^−^ + 2OH^−^ (1)

NO_3_^−^ + 7H_2_O + 8e^−^ → NH_4_^+^ + 10OH^−^ (2)

Both reactions lead to an increase in hydroxide OH^−^ ions in the solution, which then start to react with the manganese Mn^2+^ and cobalt Co^2+^ ions already present in the synthesis solution. The interaction of the OH^−^ with Mn^2+^ and Co^2+^ leads to the formation of manganese Mn(OH)_2_ (Equation (3)) and cobalt Co(OH)_2_ (Equation (4)) hydroxides, respectively.

Mn^2+^ + 2OH^−^ → Mn(OH)_2_ (3)

Co^2+^ + 2OH^−^ → Co(OH)_2_ (4)

Co(OH)_2_ + OH^−^ → CoOOH + H_2_O + e^−^ (5)

In the presence of the hydroxide ions, Co^2+^ oxidises further to Co^3+^, forming cobalt (oxy)hydroxide CoOOH (Equation (5)). The formation of (oxy)hydroxides was also observed by others [13,61].

Thus, the final form of the as-deposited Mn-Co film on nickel may consist of a mixture of Mn(OH)_2_ and Co(OH)_2_, Co_1−x_Mn_x−_(OH)_2_, Ni(OH)_2_ hydroxides, and CoOOH (oxy)hydroxides, which can be both in crystalline and amorphous phases (see Figure 2a). It should be noted that other combinations of metallic oxides/hydroxides in the deposited film are also possible.

The alkaline post-treatment of the as-deposited Mn-Co film on nickel foam results in the changing of the film structure. In the presence of a strongly alkaline environment, there is a partial oxidation of Co^2+^ ions into Co^3+^, which leads to the formation of a higher amount of cobalt (oxy)hydroxide according to Equation (5).

Besides this, the treatment of the sample with KOH also induces the formation of Mn^3+^, which was not present in the as-deposited film. Thus, the possible final form of the Mn-Co film on nickel after soaking in 1 M KOH consists of the same elements as the as-deposited film but with a higher content of Co^3+^ and with the presence of Mn^3+^, which indicates the formation of CoOOH and MnOOH, so the typical LDH structure of the film.

### 3.4. OER Performance of Mn-Co/Ni-Based Electrocatalysts

**Figure 5:**
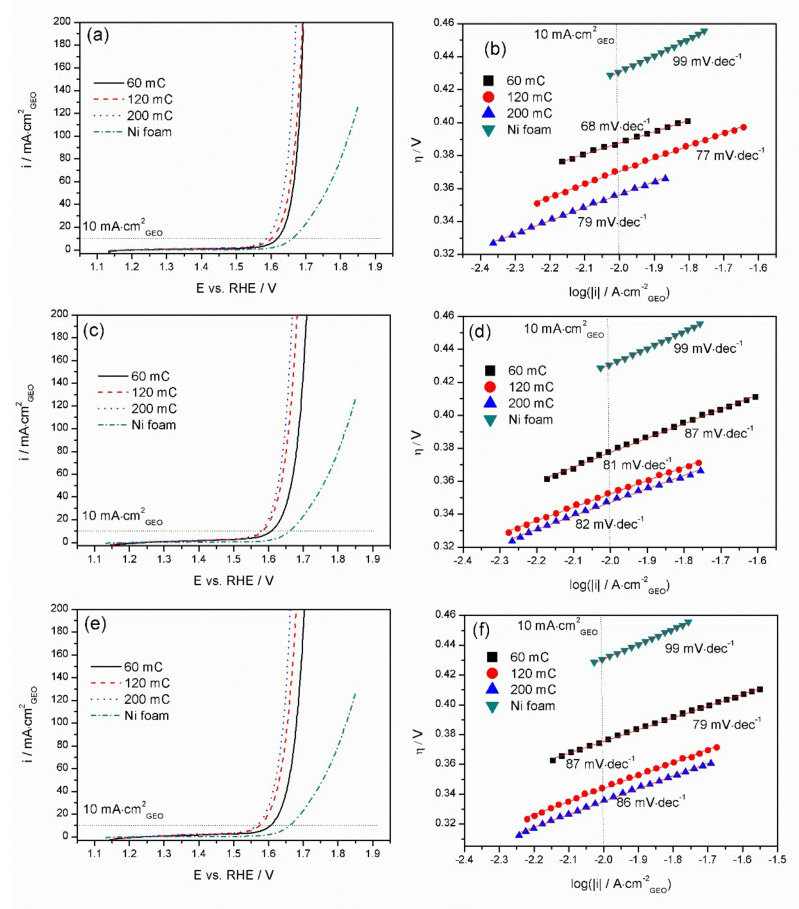
Linear sweep voltammetry profiles and corresponding Tafel plots for Mn-Co film synthesised in the presence of (**a**,**b**) Mn/Co 2 mM/4 mM, (**c**,**d**) 2 mM/6 mM, and (**e**,**f**) 2 mM/8 mM on nickel foam measured in Ar-purged 1 M KOH.

The electrocatalytical activity of Mn-Co films on nickel substrates in the OER was investigated electrochemically by linear sweep voltammetry (LSV) in an Ar-purged 1 M KOH electrolyte solution using a 5 mV·s^−1^ scan rate. All the current values were normalised by the geometric area of the nickel electrode. Figure 5 presents the LSV and corresponding Tafel plots investigated for the Mn-Co films on Ni foam synthesised in solutions of Mn/Co 2 mM/4 mM (a, b), 2 mM/6 mM (c, d), and 2 mM/8 mM (e, f) for different deposition charges. 

**Figure 6:**
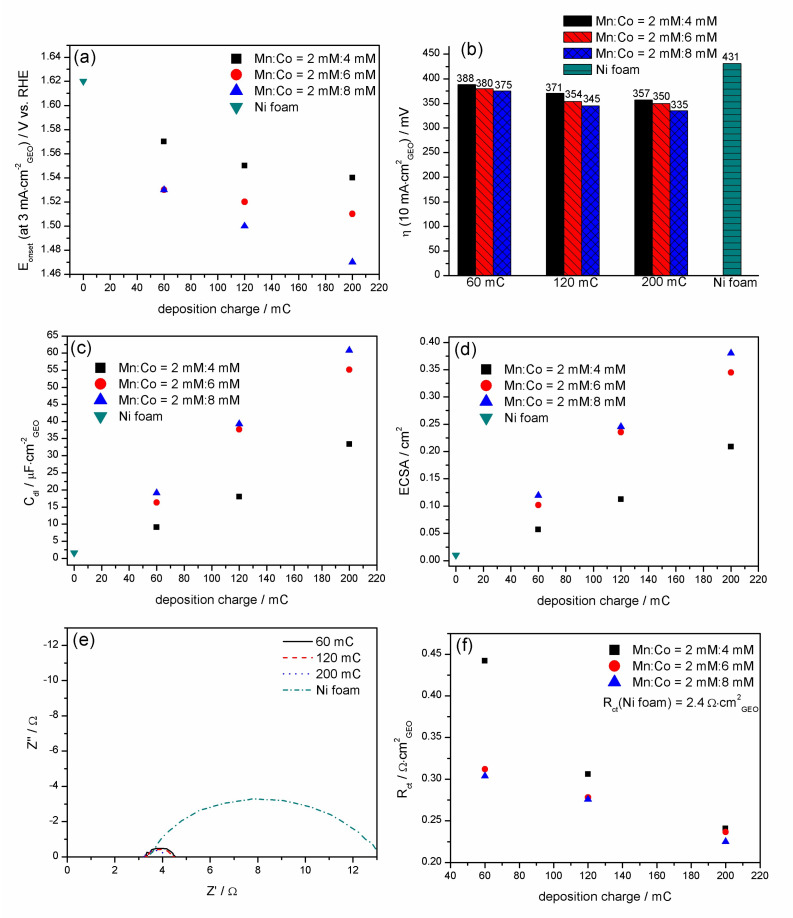
Evolution of the (**a**) onset potential, (**b**) overpotential determined at 10 mA·cm^−2^, (**c**) double-layer capacitance, (**d**) electroactive surface area (ECSA), (**e**) electrochemical impedance spectra (EIS) measured at 0.7 V vs. Ag/AgCl, and (**f**) R_ct_ of the oxygen evolution reaction (OER) as a function of the deposition charge for the Mn-Co film synthesised under different conditions on nickel foam.

The LSV graphs show that in each studied case, nickel coated with Mn-Co revealed higher electrocatalytic properties for oxygen evolution compared to bare nickel foam, which was also assessed by the evolution of the onset potential (E_onset_) and overpotential (η) of the oxygen evolution reaction (Figure 6a,b, respectively). 

The onset potential, which is an indication of the beginning of the oxygen evolution reaction, decreases over the deposition charge for each Mn/Co concentration ratio (Figure 6a). Moreover, the higher the content of cobalt in the Mn/Co concentration ratio in the synthesis solution, the lower the E_onset_. The same trend was observed in the case of overpotential, the evolution of which is presented in Figure 6b. It should be noted that the overpotential of the OER was determined here at a current density of 10 mA·cm^−2^, which is considered in the literature to be the most relevant value for solar fuel synthesis [50]. The lowest E_onset_ and η(10 mA·cm^−2^_geo_) of 1.47 V and 335 mV vs. RHE, respectively, were obtained for the Mn-Co film synthesised in the presence of Mn/Co 2 mM/8 mM for 200 mC. For comparison, the E_onset_ and η(10 mA·cm^−2^_geo_) for bare nickel foam were determined to be 1.62 V and 431 mV vs. RHE, respectively. Moreover, the OER overpotential for commercial IrO_2_ was determined in the literature to be 339 mV at 10 mA·cm^−2^_geo_ in 1 M KOH [62]. The high electrocatalytic properties of the Mn-Co film synthesised under such conditions can be related to its optimised composition, which was characterised by a high specific surface area (Figure 4a) and porosity (Figure 4b) in comparison to those of the rest of the studied samples.

Because the thicker film with the higher content of cobalt versus manganese revealed the highest electrocatalytic properties for the OER, a Mn-Co film was also electrosynthesised in the presence of Mn/Co 2 mM/8 mM for 400 mC and in the presence of a higher cobalt content, i.e., Mn/Co 2 mM/12 mM for 200 mC. The LSV results showed that further changes of the deposition charge and Mn/Co concentration ratio did not also influence the catalytic properties for the OER (data not shown here).

To assess the OER mechanism, corresponding Tafel plots for bare Ni and Mn-Co films on Ni foam electrodeposited in the presence of Mn/Co 2 mM/4 mM, 2 mM/6 mM, and 2 mM/8 mM were investigated, and are presented in Figure 5b,d,f, respectively. The Tafel slopes for Ni foam were determined to be 99 mV·dec^−1^, which is similar to those obtained in 1 M KOH in the literature [63,64]. The slopes obtained for the Mn-Co film synthesised under different conditions reveal similar values in the range of 68–86 mV·dec^−1^, which suggests that the materials exhibit similar mechanisms or pathways of OER catalysis [50]. A lower Tafel slope means fast kinetics in the OER [24]. The Mn-Co film electrodeposited potentiostatically from an aqueous solution of cobalt and manganese nitrates significantly reduces such a parameter compared to bare nickel foam. Coating the metal with the Mn-Co films resulted in a modification of the nature of the active sites and thus the rate-determining step, leading to a more efficient OER [37].

To explore the intrinsic activity of the catalysts, the electrochemical active surface areas (ECSA) of Mn-Co/Ni were evaluated from the electrochemical double-layer capacitance (C_dl_). Figure 6c,d present the evolution of C_dl_ and the corresponding ECSA, respectively, as a function of the deposition charge for differently synthesised Mn-Co films on nickel foam. The graphs clearly show an increase in C_dl_ with both the deposition charge and content of cobalt with respect to manganese in the synthesis solution (Figure 6c). The trend indicates that Mn-Co films on nickel foam prepared in the presence of a higher cobalt content and with a higher deposition charge exhibit a higher electroactive surface area. Here, it should also be noted that the value of the electrochemical surface area may not be identical to the surface area obtained from nitrogen adsorption (BET) (Figure 4a) [50]. The highest ECSA of 0.38 cm^2^ was obtained for the Mn/Co film synthesised in the solution of Mn/Co 2 mM/8 mM for 200 mC, while the lowest ECSA of 0.05 cm^2^ was obtained for the catalyst prepared in the presence of Mn/Co 2 mM/4 mM for 60 mC. All of the studied catalyst materials exhibited a higher surface area compared to bare nickel foam (C_dl_ = 1.6 μF·cm^−2^_geo_, ECSA = 0.01 cm^2^).

In order to investigate the kinetics of the OER process, EIS measurements of the Mn-Co samples were performed in Ar-purged 1 M KOH at 0.7 V vs. Ag/AgCl. Figure 6e presents examples of the Nyquist plots for Mn-Co films synthesised in the presence of Mn/Co 2 mM/8 mM for different deposition charges. The graphs clearly show the change of the shape of the EIS spectra after coating the substrate with the catalyst. In order to study the evolution of R_ct_ over the deposition charge in more detail, the EIS spectra were fitted with a simple Randles circuit with the solution resistance Rs, the charge transfer resistance R_ct_, and the constant phase element CPE (Figure 6e inset). The results indicate that the lowest R_ct_ of 0.22 Ω·cm^2^ is obtained for the catalyst synthesised in the presence of Mn/Co 2 mM/8 mM for 200 mC (Figure 6f), which is ∼11 times lower compared to that of bare nickel foam. A lower charge transfer resistance indicates much faster reaction rates for the OER. The acquired data are in agreement with the previous E_onset_, η(10 mA·cm^−2^_geo_), BET surface area, C_dl_, and ECSA analyses.

Additionally, the electrocatalytic parameters of the Mn-Co film synthesised in the presence of only cobalt nitrates (E_onset_ = 1.55 V, η(10 mA·cm^−2^_geo_) = 375 mV) or in the solution with the higher content of manganese with respect to cobalt (E_onset_ = 1.54 V, η(10 mA·cm^−2^_geo_) = 375 mV) for 200 mC were determined based on the LSV curves (Figure S7, Supplementary content). The results reveal their worse electrocatalytic properties for the OER compared to the Mn-Co film synthesised in a solution of Mn/Co 2 mM/4 mM, 2 mM/6 mM, or 2 mM/8 mM for 200 mC (Figure 6a,b). In spite of this, their performance is still much better in comparison to that of the bare nickel foam. This indicates that either too high a concentration content of manganese with respect to cobalt or a lack of manganese in the synthesis solution inhibits the electrocatalytic activity of the material in oxygen evolution. Only an appropriate concentration ratio of manganese to cobalt with a higher content of the latter induces high electrocatalytic properties of the material for the OER. Such findings might be due to the high electroactive surface area of the catalyst resulting from the nanosheet-like structure, which is created only in the presence of both manganese and cobalt oxides/hydroxides in a certain concentration ratio.

For comparison, LSV of the Mn-Co film electrodeposited on nickel foil was also performed (Figure S8, Supplementary content). The film deposited for Qd ≥ 120 mC was characterised by poor adhesion to the foil and progressive detachment from the substrate during the LSV measurements. The η(10 mA·cm^−2^_geo_) was determined to be 394 mV and 381 mV for 60 mC and 120 mC, respectively, which was higher compared to that for the Mn-Co deposited on nickel foam. The difference in the catalytic OER properties can be related to both the poor adhesion of the catalyst to the nickel foil and to the flat form of the nickel substrate. Probably, nickel in the form of a foam reveals a much more desirable structure for OER, which was also noticed by others [48].

**Figure 7:**
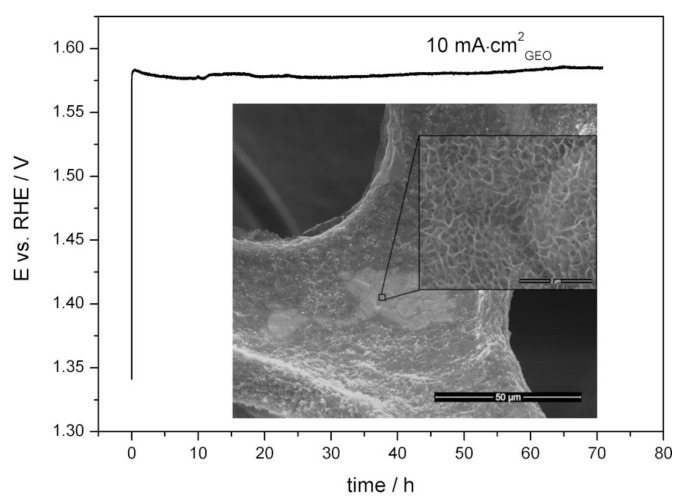
Chronopotentiometric curve recorded during the stability test of the Mn-Co film on nickel foam synthesised in a solution of Mn/Co 2 mM/8 mM for 200 mC measured in Ar-purged 1 M KOH.

Long-term stability is another key parameter for the practical application of an OER catalyst [6]. Figure 7 presents the chronopotentiometric graph recorded during the stability test of the Mn-Co film on nickel foam synthesised in a solution of Mn/Co 2 mM/8 mM for 200 mC measured in 1 M KOH by applying a current density of 10 mA·cm^−2^_geo_.

The measured potential response deviated around 1.56–1.57 V vs. RHE for 70 h, which corresponds to an overpotential of 330–340 mV. The morphology of the Mn-Co film after the stability test did not change (inset of Figure 7). There was no delamination of the catalyst from the nickel substrate after removing it from the solution. All of these indicate a high electrocatalytic stability of the proposed material during the active oxygen evolution process.

The Mn-Co films electrodeposited on nickel foam synthesised in the presence of Mn(NO_3_)_2_·4H_2_O and Co(NO_3_)_2_·6H_2_O proposed in this work reveal promising electrocatalytic properties for the OER compared to bare nickel foam. The excellent catalytic properties can be attributed to several factors. First of all, the structure of the film consists of different kinds of mixed Mn-Co oxides/hydroxides and (oxy)hydroxides (Figure 2), which provide different possibilities of multiple redox reactions of the combined Mn-Co with hydroxide ions, resulting in enhanced catalytic activity for the OER. For comparison, the performance of the catalyst based only on cobalt oxides/hydroxides was much worse, which could be seen as both a much higher OER onset potential and overpotential (Figure S7, Supplementary content). Secondly, the morphology of the Mn-Co films was characterised by interconnected nanoflakes forming a porous interconnected 3D network (Figure 1b–d), which is desirable for ion exchange reactions. Such a structure is only formed when both Mn and Co compounds are present in the synthesis solution. This indicates that both elements significantly influence the final structure of the catalyst. The SEM results also showed that a higher amount of Co nitrates in the synthesis solution did not further influence the morphology of the deposit (Figure S4, Supplementary content). On the other hand, it was shown that both C_dl_ and ECSA increased with a higher content of cobalt in the Mn/Co ratio, which in turn resulted in a lower η(10 mA∙cm^−2^_geo_), E_onset_, and R_ct_ of the OER of the catalyst. This may indicate that the introduction of a higher amount of cobalt in the catalyst structure provides a greater number of active sites responsible for an effective catalytic reaction. Therefore, it can be suggested that Co compounds in the catalyst structure, especially the highly active crystalline β-form of Co(OH)_2_, are mainly responsible for the enhancement of the catalytic properties of the electrode. The strong electrocatalytic properties of β-Co(OH)_2_ were also observed by other authors [65].

The strongest electrocatalytic properties for the OER among the studied catalysts were found for the Mn-Co film electrodeposited on nickel foam in an aqueous solution of 2 mM Mn(NO_3_)_2_·4H_2_O and 8 mM Co(NO_3_)_2_·6H_2_O for 200 mC. The catalyst was characterised by an E_onset_ of 1.47 V vs. RHE and η(10 mA·cm^−2^_geo_) of 335 mV, which indicates much higher catalytic performance for the OER in comparison to the other Mn-Co-based catalysts synthesised by chemical methods [15,62,63]. For example, the Mn-Co-based catalyst synthesised chemically was characterised by a η(10 mA·cm^−2^_geo_) of 500–600 mV in 0.1 M KOH [15]. In other work, chemically fabricated Mn-Co oxide [66] or Mn-Co oxide doped with Ce [67] exhibited η(10 mA·cm^−2^_geo_) of 450 mV and 390 mV, respectively, in 1 M KOH.

The Mn-Co-based catalyst evaluated in this work is also one of the best Mn- and/or Co-based catalysts synthesised electrochemically for the OER available in the literature (Table S1, Supplementary content). It also exhibits a lower overpotential compared to commercial IrO_2_ (339 mV at 10 mA·cm^−2^_geo_) determined in 1 M KOH [62]. The Mn-Co film synthesised under such conditions revealed the highest specific surface area (BET) and electrochemical surface area (ECSA) compared to the rest of the studied catalysts. The higher surface area provides a greater number of active sites, which allows for more efficient reactions with OH^−^. The results indicate that the surface area of the catalyst has a huge impact on the OER activity. The superior stability of the Mn-Co film on nickel foam can be related not only to the type of structure and morphology but also to the lack of a polymer binder. The latter was provided by the choice of electrochemical deposition as a synthesis method, which allowed for the direct deposition of the catalyst on nickel foam providing high adhesion of the film to the substrate.

## 4. Conclusions

A Mn-Co-based film has been successfully directly deposited on nickel foam during an electrochemical deposition process in the presence of only Mn(NO_3_)_2_·4H_2_O and Co(NO_3_)_2_·6H_2_O. A SEM analysis showed that the morphology of the Mn-Co film on nickel foam was characterised by an interconnected 3D nanoflake structure with high porosity. XRD and XPS analyses showed that the as-deposited catalysts consisted mainly of oxides/hydroxides and/or (oxy)hydroxides based on Mn^2+^, Co^2+^, and Co^3+^. The alkaline treatment of the film in 1 M KOH resulted in the partial oxidation of the Co^2+^ to Co^3+^ and the creation of Mn^3+^, leading to the formation of Mn-Co (oxy)hydroxides. Moreover, the XRD and TEM analyses showed the formation of crystalline Co(OH)_2_ with a hexagonal platelet-like shape structure. These all indicate that the final form of the catalyst is based on the LDH structure, which is highly desirable for efficient OER performance.

The electrodeposited Mn-Co film on nickel foam was found to be a very promising electrocatalyst for the OER. The catalytic performance was dependent on the concentration of Mn versus Co in the synthesis solution and on the deposition charge. The optimised catalyst was obtained for Mn/Co 2 mM/8 mM for the deposition time limited by a charge of 200 mC. It was characterised by a specific surface area of 10.5 m^2^·g^−1^, a pore volume of 0.0042 cm^3^·g^−1^, and high electrochemical stability with an overpotential deviation around 330–340 mV at 10 mA·cm^−2^_geo_ for 70 h.

## Figures and Tables

**Table 1:** The percentage content of certain elements in the as-deposited Mn-Co film and the film after alkaline treatment determined based on the XPS (surface concentration, error ≤ 5%).

Element	As-Deposited Film	Film after Alkaline Treatment
Co^2+^	79%	50%
Co^3+^	21%	50%
Mn^2+^	100%	Difficult to determine
Mn^3+^	0%	Difficult to determine

## Supplementary Materials

The following are available online at https://www.mdpi.com/1996-1944/13/11/2662/s1. Figure S1: Cyclic voltammograms recorded during sweeping the potential from 0.15 to 0.25 V vs. Ag/AgCl with different scan rates in an aqueous solution of 1 M KOH for Mn-Co film synthesised in solutions of Mn/Co 2:4 mM (a), 2:6 mM (c), and 2:8 mM (e) for 200 mC. Corresponding linear approximation of the capacitive currents versus scan rate obtained from cyclic voltammograms for Mn-Co film synthesised in solutions of Mn/Co 2:4 mM (b), 2:6 mM (d), and 2:8 mM (f) for different deposition charges. Figure S2: Synthesis graphs recorded during the potentiostatic deposition of Mn/Co oxide/hydroxides at −1.1 V vs. Ag/AgCl in aqueous solutions of differently concentrated Mn(NO_3_)_2_·4H_2_O and Co(NO_3_)_2_·6H_2_O with electropolymerisation time limited by a charge of 200 mC. Figure S3: Optical microscopy image of the as-deposited and after-alkaline-treatment-in-1 M-KOH Mn-Co film on nickel foam. Figure S4: SEM images of Mn-Co film synthesised in an aqueous solution of Mn/Co 2:4 mM for 60 mC (a), 2:4 mM for 120 mC (b), and 2:4 mM 200 mC (c) and 2:6 mM 200 mC (d) on nickel foam. Figure S5: SEM images of Mn-Co film synthesised in aqueous solution of Mn/Co 2:4 mM for 60 mC (a) and 2:8 mM for 200 mC (b) on nickel foil. Figure S6: SEM images of Mn-Co film synthesised in an aqueous solution of 4 mM Co(NO_3_)_2_·6H_2_O (a) or 4 mM Mn(NO_3_)_2_·4H_2_O and 2 mM Co(NO_3_)_2_·6H_2_O on nickel foam for 200 mC. Figure S7: Linear sweep voltammetry profiles (a) and corresponding Tafel plots (b) of Mn-Co film synthesised in solutions of Mn/Co 2:4 mM, 4:2 mM, and 0:4 mM on nickel foam measured in Ar-purged 1 M KOH. Figure S8: Linear sweep voltammetry profiles of nickel foil and Mn-Co film synthesised in a solution of Mn/Co 2:8 mM for 60 and 120 mC on nickel foil. Table S1: Comparison of catalyst based on Mn and/or Co transition metals synthesised electrochemically for OER activity available in the literature.
