# Supplementary material for: The Influence of the Electrodeposition Parameters on the Properties of Mn-Co-Based Nanofilms as Anode Materials for Alkaline Electrolysers
Source: Materials (Basel). 2020 Jun 11;13(11):2662. doi: 10.3390/ma13112662 (PMC7321643; doi:10.3390/ma13112662)
Supplement: Supplementary file 1 [file materials-13-02662-s001.pdf]

materials

MDPI

Supplementary Materials

The Influence of the Electrodeposition Parameters on the Properties of Mn-Co-Based Nanofilms as Anode Materials for Alkaline Electrolysers

Karolina Cysewska [1,*], Maria Krystyna Rybarczyk [2], Grzegorz Cempura [3], Jakub Karczewski [4], Marcin Łapiński [4], Piotr Jasinski [1] and Sebastian Molin [1]

[1] Faculty of Electronics, Telecommunications and Informatics, Gdansk University of Technology, ul. Narutowicza 11/12, 80-233 Gdansk, Poland; piotr.jasinski@pg.edu.pl (P.J.); sebastian.molin@pg.edu.pl (S.M.)
[2] Chemical Faculty, Department of Process Engineering and Chemical Technology, Gdansk University of Technology, ul. Narutowicza 11/12, 80–233 Gdansk, Poland; maria.rybarczyk@pg.edu.pl
[3] Faculty of Metals Engineering and Industrial Computer Science, International Centre of Electron Microscopy for Materials Science, AGH University of Science and Technology, ul. A. Mickiewicza 30, 30-059 Krakow, Poland; cempura@agh.edu.pl
[4] Faculty of Applied Physics and Mathematics, Gdansk University of Technology, ul. Narutowicza 11/12, 80–233 Gdansk, Poland; jakkarcz@pg.edu.pl (J.K.); marcin.lapinski@pg.edu.pl (M.Ł.)
* Correspondence: karolina.cysewska@pg.edu.pl

Received: 21 May 2020; Accepted: 10 June 2020; Published: 11 June 2020

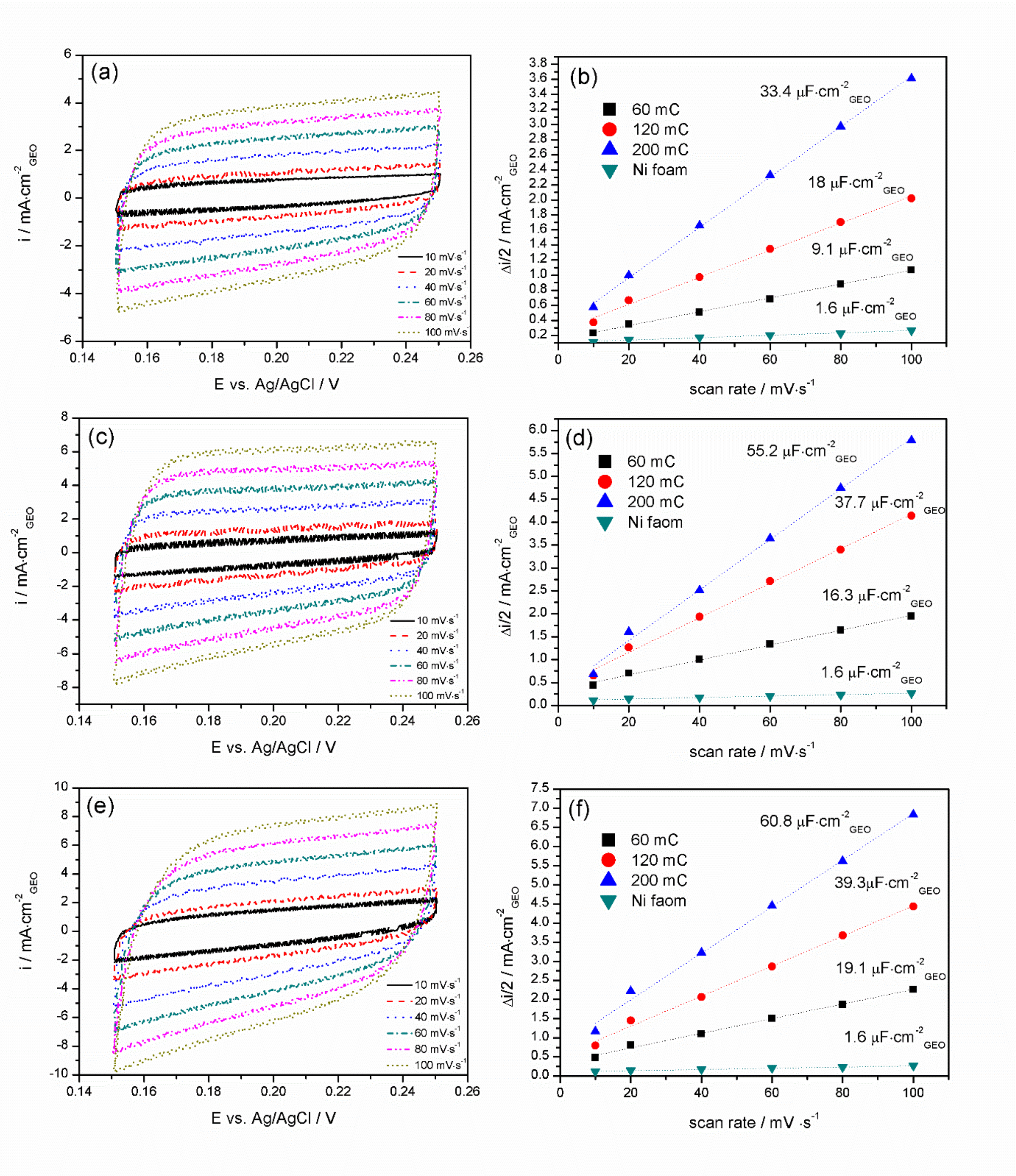

Figure S1. Cyclic voltammograms recorded during sweeping the potential from 0.15 to 0.25 V vs. Ag/AgCl with different scan rates in aqueous solution of 1 M KOH for Mn-Co film synthesized in solution of Mn:Co 2:4 mM (**a**), 2:6 mM (**c**) and 2:8 mM (**e**) for 200 mC. Corresponding linear approximation of the capacitive currents versus scan rate obtained from cyclic voltammograms for Mn-Co film synthesized in solution of Mn:Co 2:4 mM (b), 2:6 mM (**d**) and 2:8 mM (**f**) for different deposition charge.

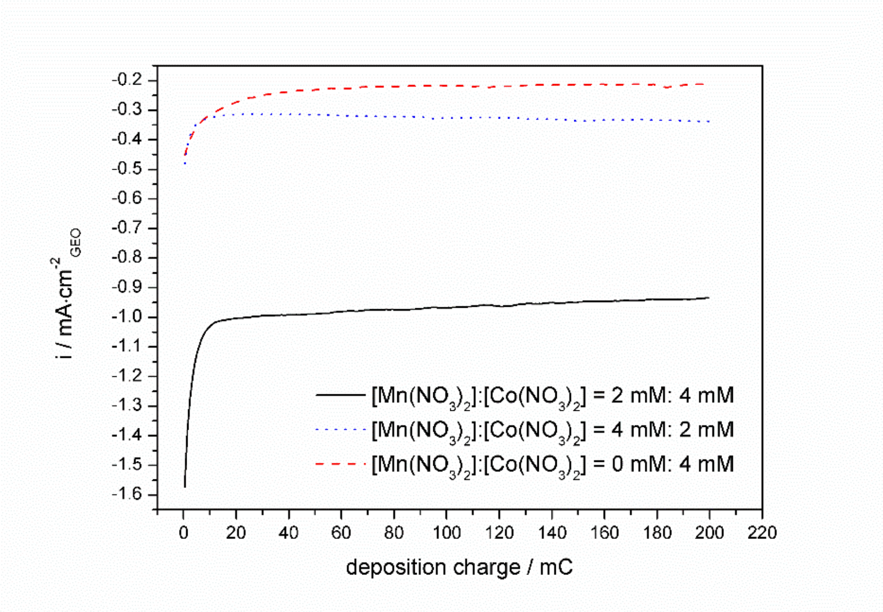

Figure S2. Synthesis graphs recorded during the potentiostatic deposition of Mn/Co oxide/hydroxides at −1.1 V vs. Ag/AgCl in aqueous solution of differently concentrated $Mn(NO_3)_2 \cdot 4H_2O$ and $Co(NO_3)_2 \cdot 6H_2O$ with electropolymerization time limited by a charge of 200 mC.

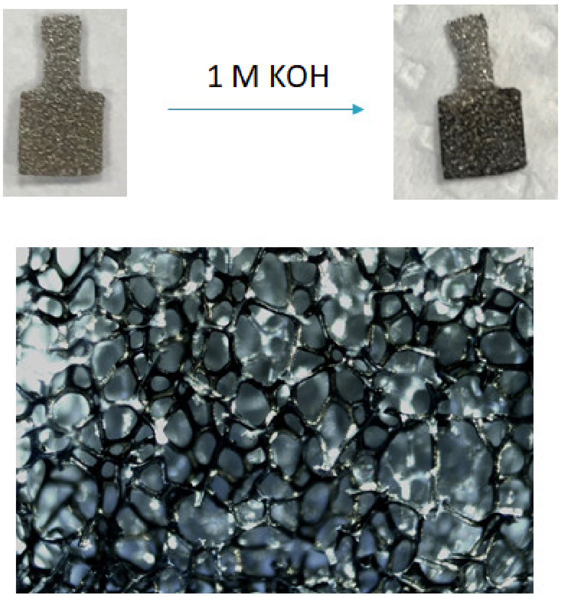

Figure S3. Optical microscopy image of the as-deposited and after alkaline treatment in 1 M KOH Mn-Co film on nickel foam.

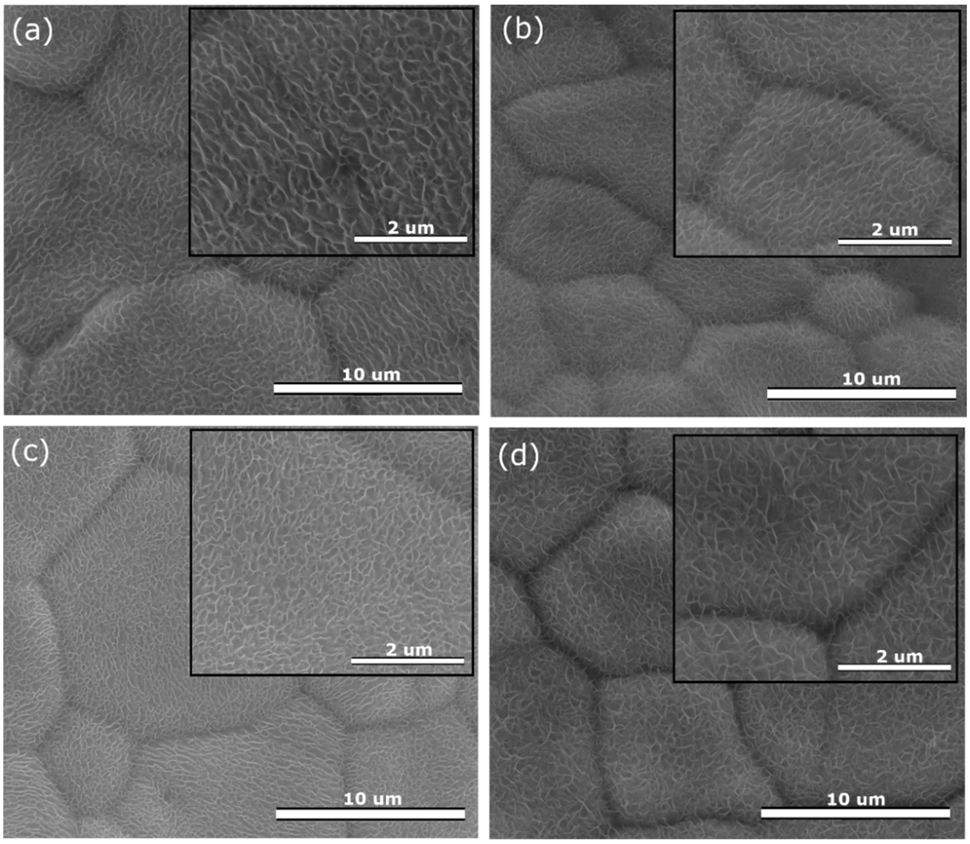

Figure S4. SEM images of Mn-Co film synthesized in aqueous solution of Mn:Co 2:4 mM for 60 mC (**a**), 2:4 mM for 120 mC (**b**), 2:4 mM 200 mC (**c**) and 2:6 mM 200 mC (**d**) on nickel foam.

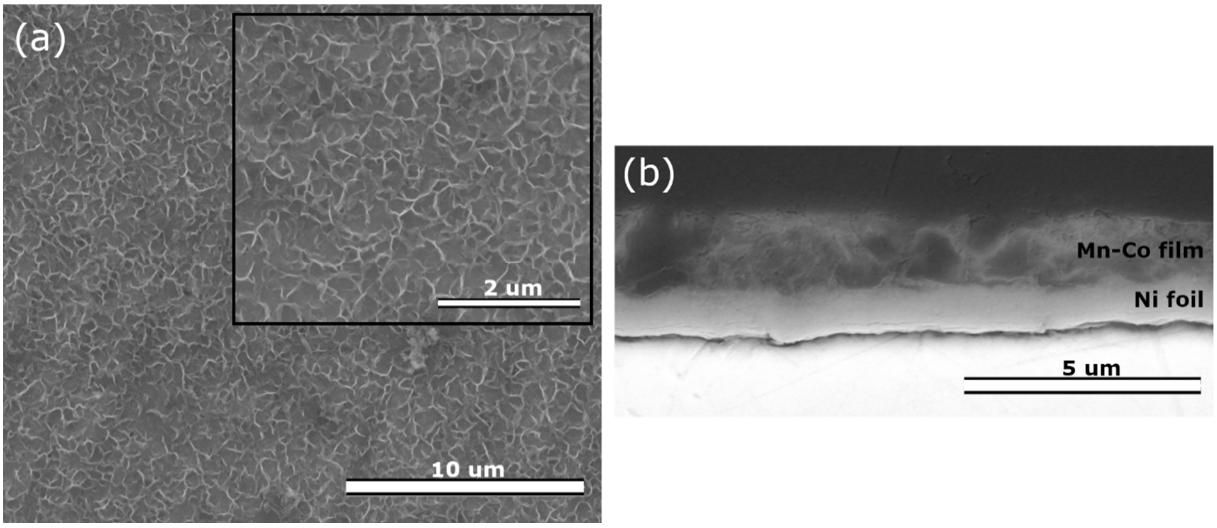

Figure S5. SEM images of Mn-Co film synthesized in aqueous solution of Mn:Co 2:4 mM for 60 mC (**a**) and 2:8 mM for 200 mC (**b**) on nickel foil.

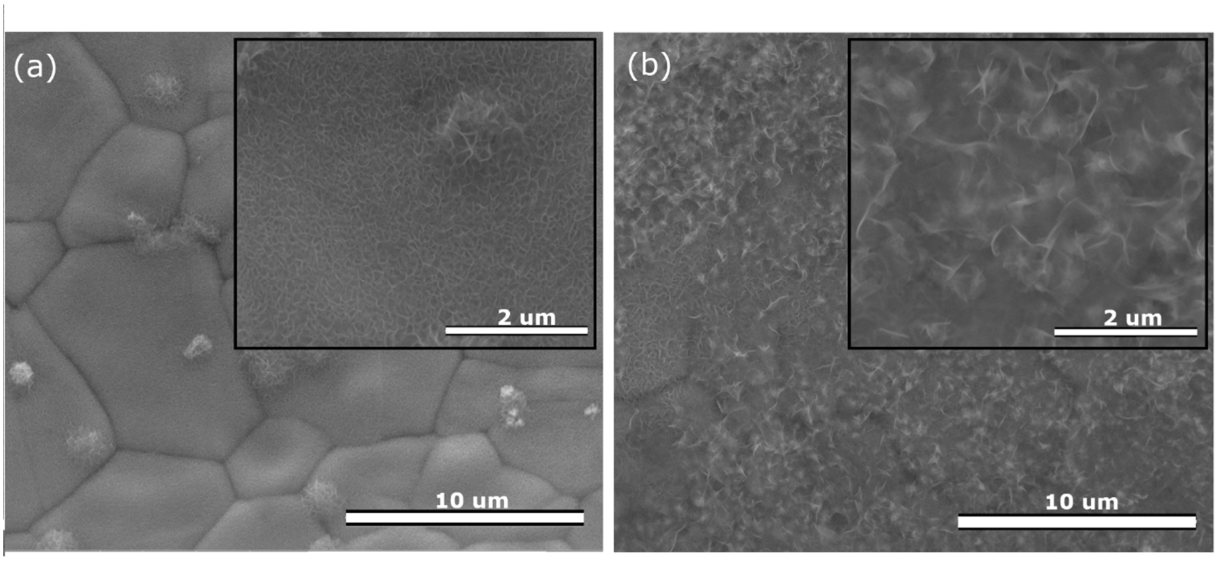

Figure S6. SEM images of Mn-Co film synthesized in aqueous solution of **(a)** 4 mM $Co(NO_3)_2{\cdot}6H_2O$ or **(b)** 4 mM $Mn(NO_3)_2{\cdot}4H_2O$ and 2 mM $Co(NO_3)_2{\cdot}6H_2O$ on nickel foam for 200 mC

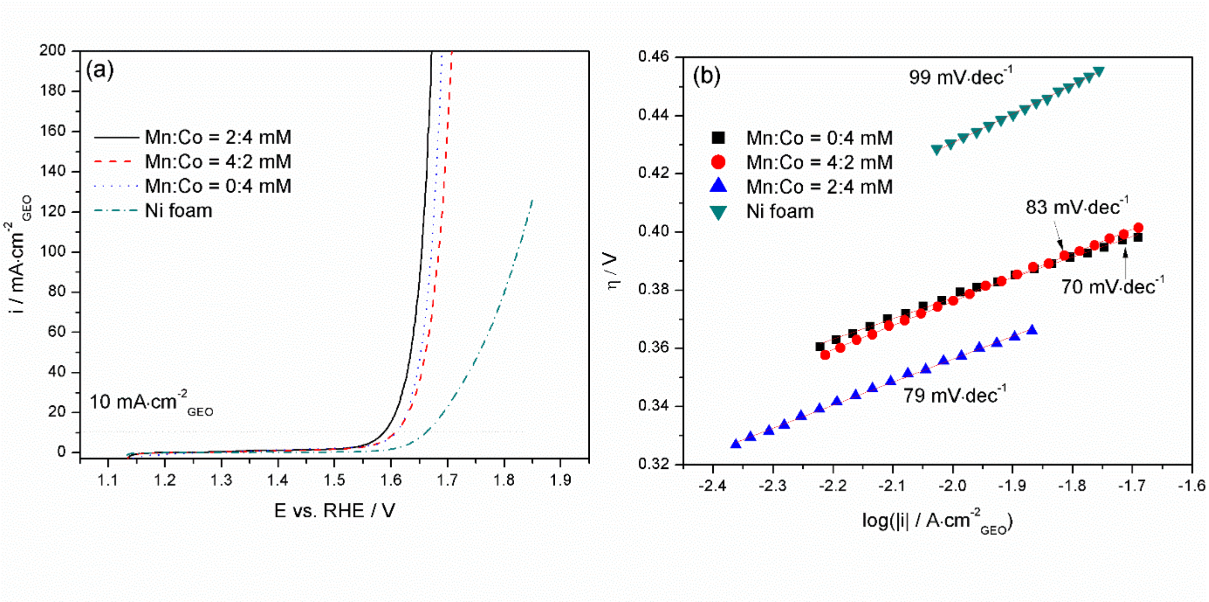

Figure S7. Linear sweep voltammetry profiles (**a**) and corresponding Tafel plots (**b**) of Mn-Co film synthesized in solution of Mn:Co 2:4 mM, 4:2 mM and 0:4 mM on nickel foam measured in Ar-purged 1 M KOH. .

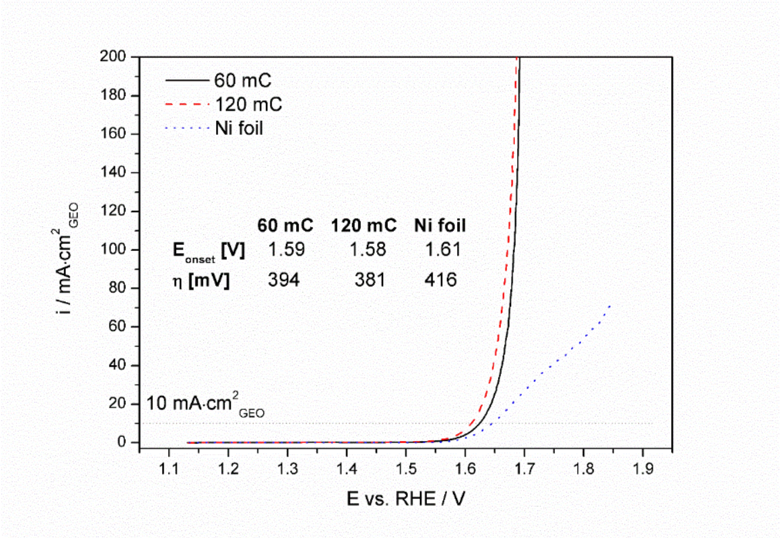

Figure S8. Linear sweep voltammetry profiles of nickel foil and Mn-Co film synthesized in solution of Mn:Co 2:8 mM for 60 and 120 mC on nickel foil.

Table S1. Comparison of catalyst based on Mn and/or Co transition metals synthesized electrochemically for OER activity available in the literature.

catalyst	substrate	solution	E_{onset} / V vs. RHE	η (10 mA·cm$^{-2}_{geo}$)/ mV	Ref.
Mn-Co	Ni foam	1 M KOH	1.47	335	This work
CoMn-LDH	carbon	0.1 M KOH	-	258	[1]
MnO_2	carbon	0.1 M KOH	-	424	[1]
MnO_x-573K	F:SnO2	1 M KOH	-	570 at 20 mA·cm$^{-2}_{geo}$	[2]
Mn_3O_4	F:SnO2	1 M KOH	-	570	[2]
Co_3O_4	Ni foil	0.1 M KOH	1.58	530	[3]
Co_3O_4	SS	1 M KOH	-	603 at 100 mA·cm$^{-2}_{geo}$	[4]
Co_3O_4	Pt	1 M KOH	-	410	[5]
$Ni_{0.6}Co_{2.4}O_4$	Ni foil	0.1 M KOH	1.57	-	[3]
$Zn_xCo_{3-x}O_4$	Au	1 M NaOH	-	330	[6]
$ZnCo_2O_4$	Ni foam	1 M KOH	-	390	[5]

References

1. Yan, F.; Guo, D.; Kang, J.; Liu, L.; Zhu, C.; Gao, P., Zhang, X.; Chen, Y. Fast fabrication of ultrathin CoMn LDH nanoarray as flexible electrode for water oxidation. *Electrochim. Acta* **2018**, *283*, 755–763.
2. Ramírez, A.; Hillebrand, P.; Stellmach, D.; May, M.M.; Bogdanoff, P.; Fiechter, S. Evaluation of MnO_x, Mn_2O_3, and Mn_3O_4 electrodeposited films for the oxygen evolution reaction of water. *J. Phys. Chem. C* **2014**, *118*, 14073–14081.
3. Lambert, T.N.; Vigil, J.A.; White, S.E.; Davis, D.J.; Limmer, S.J.; Burton, P.D.; Coker, E.N.; Beechem, T.E.; Brumbach, M.T. Electrodeposited $Ni_xCo_{3-x}O_4$ nanostructured films as bifunctional oxygen electrocatalysts. *Chem. Commun.* **2015**, *51*, 9511–9514.
4. Wu, L.K.; Hu, J.M. A silica co-electrodeposition route to nanoporous Co_3O_4 film electrode for oxygen evolution reaction. *Electrochim. Acta* **2014**, *116*, 158–163.
5. Kim, T.W.; Woo, M.A.; Regis, M.; Choi, K.S. Electrochemical synthesis of spinel type $ZnCo_2O_4$ electrodes for use as oxygen evolution reaction catalysts. *J. Phys. Chem. Lett.* **2014**, *5*, 2370–2374
6. Han, S.; Liu, S.; Wang, R.; Liu, X.; Bai, L.; He, Z. One-step electrodeposition of nanocrystalline $Zn_xCo_{3-x}O_4$ films with high activity and stability for electrocatalytic oxygen evolution. *Acs Appl. Mater. Interfaces* **2017**, *9*, 17186–17194

© 2019 by the authors. Submitted for possible open access publication under the terms and conditions of the Creative Commons Attribution (CC BY) license (http://creativecommons.org/licenses/by/4.0/).
